# Angiopoietin-Like Protein 8/Leptin Crosstalk Influences Cardiac Mass in Youths With Cardiometabolic Risk: The BCAMS Study

**DOI:** 10.3389/fendo.2021.788549

**Published:** 2022-01-25

**Authors:** Dongmei Wang, Dan Feng, Yuhan Wang, Peiyu Dong, Yonghui Wang, Ling Zhong, Bo Li, Junling Fu, Xinhua Xiao, John R. Speakman, Ming Li, Shan Gao

**Affiliations:** ^1^Department of Endocrinology, National Health Commission (NHC) Key Laboratory of Endocrinology, Peking Union Medical College Hospital, Peking Union Medical College and Chinese Academy of Medical Sciences, Beijing, China; ^2^Department of Endocrinology, Beijing Chaoyang Hospital, Capital Medical University, Beijing, China; ^3^Center for Energy Metabolism and Reproduction, Shenzhen Institutes of Advanced Technology, Chinese Academy of Sciences, Shenzhen, China; ^4^State Key Laboratory of Molecular Developmental Biology, Institute of Genetics and Developmental Biology, Chinese Academy of Sciences, Beijing, China

**Keywords:** angiopoietin-like protein 8, leptin, left ventricular mass, youth, lipids

## Abstract

**Objectives:**

The link between excess adiposity and left ventricular hypertrophy is multifaceted with sparse data among youths. Given that adipokines/hepatokines may influence lipid metabolism in myocardium, we aimed to investigate the relation of the novel hepatokine angiopoietin-like protein 8 (ANGPTL8) and other adipokines with cardiac structure in a cohort of youths and explore to what extent these adipokines/hepatokines affect cardiac structure through lipids.

**Methods:**

A total of 551 participants (aged 15-28 years) from the Beijing Child and Adolescent Metabolic Syndrome Study (BCAMS) cohort underwent echocardiographic measurements plus a blood draw assayed for five adipokines/hepatokines including adiponectin, leptin, retinol binding protein 4, fibroblast growth protein 21 and ANGPTL8.

**Results:**

Both ANGPTL8 (β = -0.68 g/m^2.7^ per z-score, *P*= 0.015) and leptin (β = -1.04 g/m^2.7^ per z-score, *P*= 0.036) were significantly inversely associated with left ventricular mass index (LVMI) independent of classical risk factors. Total cholesterol and low-density lipoprotein cholesterol significantly mediated the ANGPTL8–LVMI association (proportion: 19.0% and 17.1%, respectively), while the mediation effect of triglyceride on the ANGPTL8–LVMI relationship was strongly moderated by leptin levels, significantly accounting for 20% of the total effect among participants with higher leptin levels. Other adipokines/hepatokines showed no significant association with LVMI after adjustment for body mass index.

**Conclusions:**

Our findings suggest ANGPTL8, particularly interacting with leptin, might have a protective role in cardiac remodeling among youths with risk for metabolic syndrome. Our results offer insights into the pathogenesis of the cardiomyopathy and the potential importance of tissue-tissue crosstalk in these effects.

## Introduction

A growing epidemic of overweight and obesity is affecting many countries worldwide, and the standardized prevalence of obesity in Chinese children has increased rapidly from 1.7% in 1991–1995 to 6.8% in 2011–2015 (1). Development of cardiovascular diseases could originate in early childhood, especially in those with obesity (2). Notably, individuals with obesity are frequently at risk of left ventricular hypertrophy (LVH), particularly in children and adolescents even when they are uncomplicated by hypertension or diabetes (3). LVH is an independent predictor of cardiovascular events such as heart failure and cardiovascular mortality (4). However, the links between excess adiposity and LVH are multifaceted and still not completely understood. One potentially important pathophysiological mechanism for cardiac remolding is due to obesity-induced adipokine dysregulation (5, 6).

Adipose tissue is now recognized to be a dynamic endocrine organ secreting numerous signaling proteins collectively termed adipokines, that may exert several biological actions, including those on heart and vessels (7). Accumulating evidence indicates that adipokine dysregulation was associated with excess fat accumulation in visceral and ectopic sites (e.g., heart and liver), which can contribute either directly or indirectly to the development of cardiometabolic disease through modulation of risk factors such as dyslipidemia, diabetes, hypertension and the metabolic syndrome (MS) (7, 8). Particularly, the main adipokines, such as adiponectin, leptin, retinol-binding protein 4 (RBP4), and the hepatokine fibroblast growth factor 21 (FGF21) (9), have been reported to play a role in adiposity-related cardiac dysfunction both in clinical and experimental studies. Population based studies have found that higher circulating leptin levels contributed to smaller left ventricular mass (LVM) and a lower odds ratio for the presence of LVH (10, 11), and the potential mechanism might be reducing the storage of triglyceride (TG) in the cardiomyocytes (12–14). However, as the one of the most studied adipokines in metabolic disorders, leptin showed a paradoxical association with cardiovascular diseases in clinical studies (14); while adiponectin, a well-established anti-inflammatory adipokine, protected against cardiac hypertrophy and was found to be related to LVH in several clinical studies (15, 16), but not others (11, 17). Experimental studies have shown that RBP4 promoted cardiac hypertrophy by directly stimulating cardiomyocytes or as crucial mediators inducing inflammation and oxidative stress in myocardial hypertrophic responses (18). In a community-based cohort study of individuals with mean age 40 years, plasma RBP4 was positively associated with LVM index (11). In addition, while experimental study found FGF21 protects against cardiac hypertrophy related to β-oxidation of fatty acids (FAs) in mice (19), previous clinical studies found that increased FGF21 level was associated with adverse cardiac alterations (20, 21). However, population studies of these adipokines/hepatokines with cardiac hypertrophy are limited to elderly populations, focusing on only one or two adipokines, and remain inconsistent (10, 11, 15, 16). Systematic study of these adipokine profiles and their associations to LVH in young populations is still lacking.

Recently, another novel hepatokine angiopoietin-like protein 8 (ANGPTL8), which is exclusively secreted by the liver in human (22), alternatively named as betatrophin (23), has recently been discovered as a member of the ANGPTL3/4/8 family of proteins involved in lipid metabolism *via* inhibiting lipoprotein lipase (LPL) activity (24, 25). Interestingly, ANGPTL8 is increased in the fed state to inhibit LPL activity specifically in cardiac and skeletal muscles to minimize uptake of FAs, and direct circulating triglyceride to white adipose tissue for storage (26). Thereby, dysregulation of ANGPTL8 might be involved in changes of ectopic fat deposition and cardiac structure. However, study of ANGPTL8 levels in relation to cardiac geometry has not yet been reported.

Given that all the above-noted hormones might impact lipid metabolism in the myocardium relevant to LVH, and echocardiography has been shown to be a sensitive tool for detecting the functions and abnormalities of cardiac structure (27), we aimed to systematically investigate the associations between circulating levels of these functionally prominent adipokines/hepatokines in relation to the changes of echocardiography parameters among youths with risk for MS. We hypothesized that the adipokine/hepatokine-mediated crosstalk between tissues may provide potential opportunities to improve cardiac structure and function. In addition, we also determined quantitatively the possible mediation effect of lipid profiles on the association of adipokines/hepatokines with cardiac geometry.

## Material and Methods

### Participants

Details about the design and the selection criteria of the Beijing Child and Adolescent Metabolic Syndrome (BCAMS) have been published elsewhere (28, 29). Briefly, the BCAMS study began in 2004, as a prospective cohort study of identifying cardiovascular risk factors from childhood to adulthood. Among the 19593 participants (aged 6 -18 years, 50% boys) enrolled in the study, about 4500 individuals were identified as being at a risk of cardiovascular disease due to the presence of at least one of the following risk factors: overweight defined by a body mass index (BMI) at ≥ 85th percentile; blood pressures at ≥ 90th percentile, TG of ≥ 1.7 mmol/L, total cholesterol (TC) of ≥ 5.2 mmol/L or fasting glucose of ≥ 5.6 mmol/L based on capillary blood sampling. Beginning in 2012, these at-risk individuals were recruited consecutively through various modalities for a medical examination in a clinical center at Beijing Chaoyang Hospital (28). A total of 551 participants (15-28 years, mean = 20.2 years) had complete data and were included into this analysis (**Supplementary Figure 1**). The study protocol was approved by the Ethics Committee at the Beijing Chaoyang Hospital and was in accordance with the declaration of Helsinki on ethical principles for medical research involving human subjects. Written informed consent was obtained from all patients before participation in this study. The study was registered on www.clinicaltrials.gov (NCT03421444).

### Clinical, Demographic, Anthropometric and Blood Measurements

After a minimum 10-hour overnight fast, participants came to the clinical center where demographic, laboratory, anthropometric details and blood pressures were taken as previously reported (25). BMI was calculated as weight divided by height squared. Lifestyle factors and health history information were obtained by standardized questionnaire (28, 29).

A 2-hour oral glucose tolerance test using 75g glucose load was performed. Blood glucose and lipids, including TG, TC, low-density lipoprotein cholesterol (LDL-C), and high-density lipoprotein cholesterol (HDL-C) were measured using standard methods (28). Insulin, leptin and adiponectin concentrations were measured by monoclonal antibody-based sandwich enzyme-linked immunosorbent assays (ELISA) (29–32). Insulin assay had an inter-assay CV of <9.0% and no cross-reactivity to proinsulin (<0.05%) (31). The intra-assay and inter-assay CVs were <5.4% and <8.5% for adiponectin (30), and <7.4% and <9.3% for leptin, respectively (32). FGF21 was measured by Human Quantikine ELISA Kit (R&D Systems, Inc.) with intra- and inter-assay CVs of <4.8% and <7.4%, respectively (32, 33). RBP4 was measured by ELISA kits (Dou set, R&D Systems) with intra-and inter-assay CVs of 6.2% and 8.5%, respectively (34). ANGPTL8/betatrophin levels were measured by an ELISA (WUHAN EIAAB Science; catalog number E11644 h) with intra- and inter-assay CVs of <8% and <10%, respectively (28). All samples were analyzed in duplicate. Insulin resistance was assessed by the homeostasis model assessment of insulin resistance (HOMA-IR), calculated as fasting insulin (μIU/ mL) × fasting blood glucose (mmol/L)/22.5 (35), and whole-body insulin sensitivity (Matsuda) index (ISI_Matsuda_, reflecting both hepatic and peripheral insulin sensitivity) during the OGTT calculated as ISI_Matsuda_ = 10 000/(FPG × FIns)×(G × I) (36), where G=mean serum glucose and I=mean serum insulin level.

### Echocardiography

Echocardiographic measurements were performed using previously described methods (28, 29). Non-invasive transthoracic echocardiogram was performed with a LOGIQ P5 B-mode ultrasonogram equipped with a 2.5-3.5MHz probe. All measurements were recorded and analyzed by an experienced investigator who was blinded to group, including interventricular septal diastolic thickness (IVSDT), left ventricular end-diastolic diameter (LVEDD), and left ventricular posterior wall thickness (LVPWT). LVM was calculated using the formula: LVM=0.8*{1.04*[(LVEDD+ LVPWT + IVSDT)^3^−(LVEDD)^3^]}+0.6 (37). To adjust for the influence of growth on LVM without eliminating the impact of excess adiposity, LVM was indexed to height^2.7^ (LVMI) (37, 38).

### Data Analysis

**Figure 1:**
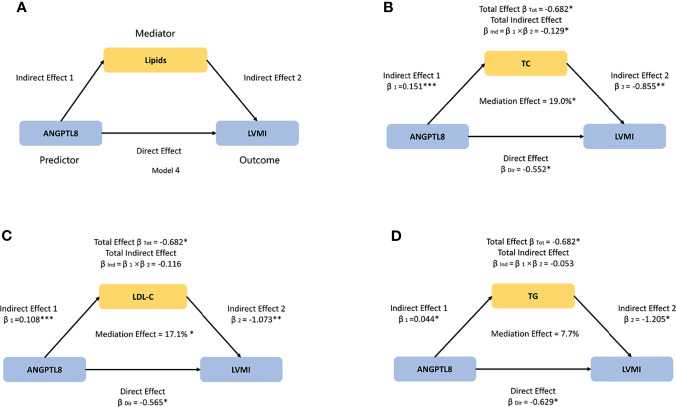
The model used to evaluate the mediation effects of lipids on ANGPTL8-LVMI association. **(A)** We hypothesized that ANGPTL8 may indirectly affect left ventricular mass index (LVMI) by regulating lipids metabolism including total cholesterol (TC), low-density lipoprotein cholesterol (LDL-C), and triglyceride (TG). **(B)** TC significantly mediated 19.0% of the effect of ANGPTL8 on LVMI. **(C)** LDL-C significantly mediated 17.1% of the effect of ANGPTL8 on LVMI. **(D)** The effect that was mediated by TG was 7.7%, although it was not significant. Mediation effects were adjusted for age, sex, body mass index, and systolic blood pressure; per z-score increment of ln-ANGPTL8. β = standardized regression coefficient; β_1_ = indirect effect 1; β_2_ = indirect effect 2; β_Ind_ = total indirect effect; β_Dir_ = direct effect; β_Tot_ = total effect; Data based on 5000 bootstrap samples. *P < 0.05; **P < 0.01; ***P < 0.001.

Database management and statistical analyses were performed using the Statistical Package for Social Sciences (SPSS 25.0 for Windows, SPSS Inc., USA). A *P*-value of <0.05 (two-side) was considered statistically significant. Continuous variables were tested for normality using the Kolmogorov-Smirnov test. Non-normal distribution values such as insulin, leptin, adiponectin, FGF21, RBP4, ANGPTL8, and TG were natural logarithmically transformed (ln-transformed) to comply with normality assumptions of the tests. Comparison between the groups was achieved using the t test or analysis of variance for continuous variables, while categorical variables were explored using the chi-square test. Multiple liner regression analyses were used to examine the relationships between adipokines/hepatokines and cardiac parameters after controlling for potential confounding factors. Parameter estimate (β) for liner regression analysis were evaluated to per z-score or standard deviation (SD) increment in each adipokine for the purpose of comparison across adipokines/hepatokines with different union of quantity. Interaction analysis was performed using linear regression models, and stratified analysis were performed when interactions effects were significant. The mediation and moderation analyses were performed using PROCESS Procedure for SPSS Release 3.3 (39). Mediation and moderation effects were evaluated with linear regression models for LVMI, adjusting for confounding factors. First, we tested whether lipids served as a mediator on the associations between adipokines/hepatokines and LVMI by using the mediation template Model 4 (**Figure 1**). Second, we tested whether an indirect effect (mediation) of ANGPTL8 on LVMI is dependent on another variable-leptin (moderation) by using a moderated mediation template Model 7 (**Figure 3**). The 95% confidence intervals (CIs) were obtained *via* 5000 bootstraps. We also reported the *P* values of the mediation results based on Sobel tests.

## Results

### Characteristics of Participants

The clinical, biochemical and echocardiographic features of the study sample are shown in **Table 1**. Of the 551 participants, 291 (52.8%) were males, with the mean age of 20.2 years. Due to the difference in adipokine/hepatokine levels in relation to sex, sex-adjusted z-scores after ln-transformation were used to provide comparability across various adipokines/hepatokines in subsequent regression analysis.

**Table 1:** Characteristics of participants.

	All	Male	Female	*P*
**Clinical characteristics**				
N	551	291	260	—
Age (year)	20.2 (2.9)	20.0 (3.0)	20.4 (2.8)	0.110
Moderate to high activity (h/week)	2.2 (1.9)	3.2 (3.8)	1.7 (2.0)	**<0.001**
Smoking, n (%)	109 (19.5%)	75 (25.5%)	34 (12.8%)	**<0.001**
Waist circumference (cm)	85.2 (14.6)	90.5 (14.6)	79.3 (12.1)	**<0.001**
BMI (kg/m^2^)	25.7 (5.7)	27.0 (5.8)	24.3 (5.3)	**<0.001**
SBP (mmHg)	115 (14)	121 (14)	108 (11)	**<0.001**
DBP (mmHg)	73 (10)	76 (10)	70 (10)	**<0.001**
Fasting blood glucose (mmol/L)	4.92 (0.69)	5.00 (0.86)	4.82 (0.41)	**0.001**
2h- blood glucose (mmol/L)	6.06 (1.84)	6.17 (2.07)	5.95 (1.54)	0.179
Fasting insulin (mIU/L) †	1.94 (0.74)	1.97 (0.75)	1.89 (0.72)	0.219
2h-insulin (mIU/L) †	3.61 (0.80)	3.55 (0.86)	3.67 (0.73)	0.108
ISI_Matsuda_ †	1.79 (0.65)	1.76 (0.69)	1.82 (0.64)	0.278
HOMA-IR †	0.41 (0.77)	0.46 (0.77)	0.35 (0.75)	0.092
Triglyceride (mmol/L) †	-0.03(0.52)	0.03(0.57)	-0.1(0.45)	**0.003**
Total cholesterol (mmol/L)	4.35 (0.92)	4.29 (0.86)	4.41 (0.99)	0.131
LDL-C (mmol/L)	2.53 (0.79)	2.56 (0.72)	2.50 (0.86)	0.371
HDL-C (mmol/L)	1.44 (0.32)	1.34 (0.28)	1.54 (0.34)	**<0.001**
hs-CRP (mg/L) †	-0.88 (1.77)	-0.73 (1.70)	-1.06 (1.84)	**0.029**
Vitamin D (ng/ml) †	2.64 (0.40)	2.76 (0.40)	2.52 (0.38)	**<0.001**
**Adipokines/hepatokines**				
Adiponectin (ug/ml) †	1.90 (0.65)	1.75 (0.72)	2.07 (0.53)	**<0.001**
Leptin (ng/ml) †	0.59 (1.05)	0.12 (1.04)	1.11 (0.78)	**<0.001**
FGF21 (pg/ml) †	4.39 (1.12)	4.39 (1.11)	4.39 (1.13)	0.893
RBP4 (ug/ml) †	2.19 (0.38)	2.24 (0.42)	2.14 (0.32)	**0.002**
ANGPTL8 (pg/ml) †	5.77 (0.37)	5.82 (0.36)	5.71 (0.37)	**<0.001**
**Cardio-parameters**				
IVSDT (cm)	0.89 (0.12)	0.93 (0.12)	0.85 (0.10)	**<0.001**
LVEDD (cm)	4.42 (0.49)	4.65 (0.45)	4.17 (0.40)	**<0.001**
LVPWT (cm)	0.89 (0.11)	0.94 (0.10)	0.84 (0.10)	**<0.001**
LVM (g)	130.33 (38.16)	150.41 (35.83)	109.31 (27.84)	**<0.001**
LVMI (g/m^2.7^)	30.87 (7.80)	33.59 (8.08)	29.07 (7.08)	**<0.001**

Data are the means (SD) or N (%). P values are for the sex differences. Values in bold are significant at P < 0.05. ^†^Ln-transformed. BMI, body mass index; SBP, systolic blood pressure; DBP, diastolic blood pressure; ISI_Matsuda_, insulin sensitivity (Matsuda) index; HOMA-IR, homeostasis model assessment for insulin resistance; LDL-C, low-density lipoprotein cholesterol; HDL-C, high-density lipoprotein cholesterol; hs-CRP, high-sensitivity c-reactive protein; FGF21, fibroblast growth factor 21; RBP4, retinol binding protein 4; ANGPTL8, angiopoietin-like protein 8; IVSDT, inter ventricular septal diastolic thickness; LVEDD, left ventricular end-diastolic diameter; LVPWT, left ventricular posterior wall thickness; LVM, left ventricular mass; LVMI, left ventricular mass index.

### Associations of Adipokines/Hepatokines With Cardio-Parameters

After adjustment for confounding factors, levels of each adipokine/hepatokine in relation to the cardiac parameter LVMI in multivariate linear regression models were presented in **Table 2**. Blood pressures were certified a conventional risk factor for LVH, therefore we considered systolic blood pressure (SBP) as a confounding factor in the regression models (40). When adipokines/hepatokines were modeled separately, higher ANGPTL8 was significantly associated with smaller LVMI (β = -0.68 g/m^2.7^ per sex-adjusted ln-ANGPTL8 z-score, *P* = 0.015) after adjustment for age, sex, BMI and SBP. When other adipokines/hepatokines were assessed together, the significant association between ANGPTL8 with LVMI remained highly significant (*P* =0.004). In addition, a significant negative association of leptin with LVMI was also evident regardless of whether the other adipokines/hepatokines were added separately or together in models (all *P*<0.05). However, as shown in **Supplementary Table 1**, adiponectin and FGF21 were found to be a negatively associated with LVM or LVMI independent of age and sex, but these significant relationships disappeared after further adjustment for BMI. Meanwhile, higher RBP4 level was associated with higher LVMI, but this association disappeared after controlling for age and sex (**Supplementary Table 1** and **Supplementary Figure 2**). Due to the novel association for ANGPTL8 and unexpectedly, this association appeared to be more significant than the classical adipokines, we next focused on further analysis for ANGPTL8. To explore whether the association between ANGPTL8 and LVMI was linear or not, ANGPTL8 levels were also categorized into sex-standardized quartiles and the group differences in cardio-parameters were compared (**Supplementary Table 2**). While there were significant differences in lipid profiles (TG, TC, and LDL-C, but not HDL-C) across the ANGPTL8 quartiles, there was a declining trend in LVEDD, LVM and LVMI with increasing quartiles of ANGPTL8 levels (*P* = 0.019, *P* = 0.008, *P* = 0.016, respectively). Additional adjustments for other traditional cardiovascular disease risk factors, including fasting blood glucose, insulin resistance, lipid profiles, hs-CRP and lifestyle factors like moderate to high activity and smoking; and further other classical adipokines including leptin, adiponectin, RBP4 and hepatokine FGF21, did not materially change the significance of the associations, while the progressive trends were observed with the increasing quartiles of ANGPTL8 levels (data not shown).

**Table 2:** Associations of adipokines/hepatokines with LVMI in multivariate linear regression.

	β (95% CI)	*P*
**Panel 1—adipokines/hepatokines modeled separately**		
Leptin (ng/ml)^†^	-1.04 (-1.88 to -0.19)	**0.036**
FGF21 (pg/ml)^†^	-0.04 (-0.65 to 0.56)	0.886
Adiponectin (μg/ml)^†^	-0.10 (-0.53 to 0.75)	0.743
ANGPTL8 (pg/ml)^†^	-0.68 (-1.23 to -0.14)	**0.015**
RBP4 (ug/ml)^†^	-0.16 (-0.59 to 0.62)	0.956
**Panel 2—adipokines/hepatokines modeled together**		
Leptin (ng/ml)^†^	-1.14 (-2.03 to -0.25)	**0.012**
FGF21 (pg/ml)^†^	-0.03 (-0.65 to 0.59)	0.924
Adiponectin (μg/ml)^†^	0.20 (-0.46 to 0.86)	0.551
ANGPTL8 (pg/ml)^†^	-0.83 (-1.38 to -0.23)	**0.004**
RBP4 (ug/ml)^†^	0.12 (0.51 to 0.75)	0.705

^^†^^Ln-transformed, per z-score increment of each ln-adipokines/hepatokines, adjusted for age, sex, body mass index, and systolic blood pressure. Values in bold are significant at P < 0.05. β, parameter estimate; CI, confidence interval; FGF21, fibroblast growth factor 21; ANGPTL8, angiopoietin-like protein 8; RBP4, retinol binding protein 4.

### The Mediation Effects of Lipids on ANGPTL8-LVMI Association

Given that ANGPTL8 has been strongly implicated in lipid metabolism, we then conducted mediation analyses to explore whether and to what extent the circulating lipids (TG, TC, and LDL-C, but not HDL-C, which were significantly different across the ANGPTL8 quartiles, **Supplementary Table 2**), contributing to linking ANGPTL8 with cardiac remodeling. Mediation effects of lipids on ANGPTL8-LVMI associations were tested for adjustment of age, sex, BMI and SBP by using Model 4 (**Figure 1A**). As shown in **Figures 1B**–**D**, the total indirect effects *via* TC, TG, or LDL-C were defined as the product of indirect effect 1 (β_1_) and indirect effect 2 (β_2_).The total effect of ANGPTL8 on LVMI was -0.682 g/m^2.7^ per ln-ANGPTL8 z-score (*P*=0.015). Circulating TC levels significantly mediated the ANGPTL8-LVMI relationship (β_Ind_ = -0.129 g/m^2.7^, 95%CI: [-0.324 to -0.026], *P*=0.031), accounting for 19.0% of the total effect (**Figure 1B**), while LDL-C levels accounted for 17.1% of the total effect of ANGPTL8 on LVMI (β_Ind_ = -0.116, 95%CI: [-0.325 to -0.014], *P*=0.036) (**Figure 1C**). However, the effect that was mediated by TG was unexpectedly not significant (P =0.167), although it accounted for 7.7% of the total effect (β_Ind_ = -0.053, 95%CI: [-0.177 to 0.024]) (**Figure 1D**). Additional adjustments for other traditional cardiovascular disease risk factors such as HOMA-IR or ISI_Matsuda_, the mediation effects were not materially changed (data not shown). Thereby we only show the results with adjustment of age, sex, BMI and SBP (**Figure 1**).

### The Moderation Effects of Leptin and the Mediation Effects of TG on ANGPTL8-LVMI Association

**Figure 2:**
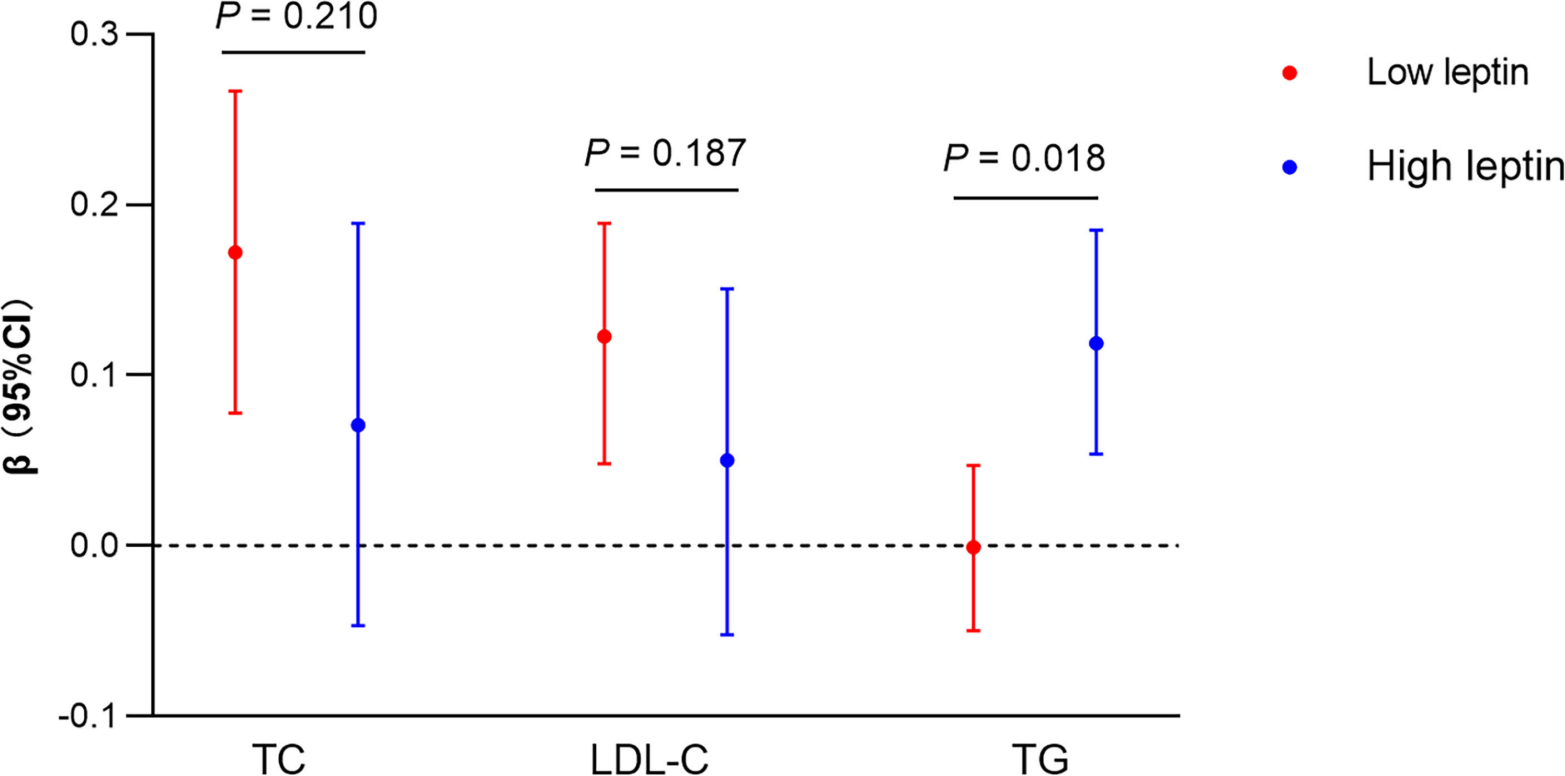
Interaction between ANGPTL8 and leptin on lipids profiles. Liner regression analysis of ANGPTL8 on lipids (TG, TC and LDL-C) stratified by high and low (above/below sex-specific median) levels of leptin. Ln-transformed for triglyceride (TG) and ANGPTL8, per z-score increment of each ln-ANGPTL8. Liner regression models and the *P* for interaction were shown adjusted for age, sex, body mass index, and systolic blood pressure. β, parameter estimate from linear regression; CI, confidence interval; TC, total cholesterol; LDL-C, low-density lipoprotein cholesterol.

Next, we sought to clarify why we did not observe a significant mediation effect of TG on the ANGPTL8-LVMI relationship. Given that previous studies have found that higher leptin levels related to more favorable measures of structure *via* promoting β-oxidation of FAs to reduce the storage of TG in the cardiomyocytes (10), and as shown in **Table 2**, like ANGPTL8, leptin replicated a negative association with LVMI in our data. We hypothesized that leptin might play a moderating role in the mediation effect of TG on the ANGPTL8-LVMI relationship. Thus, models assuming interaction between ANGPTL8 and leptin on TG levels were first adopted. As listed in **Figure 2**, ANGPTL8 and leptin showed a significant interaction effect on TG levels (*P* for interaction = 0.018), but not on other lipids. Accordingly, we further stratified individuals into 2 groups based on the sex-specific median of leptin as high/low group (above/below the sex-specific median). In these analyses ANGPTL8 was significantly associated with higher TG levels (β = 0.119 mmol/L, 95% CI [0.054, 0.185], *P* < 0.001) in the high leptin group, while no significant association was found in low leptin group (β = -0.001 mmol/L, 95%CI [-0.050, 0.047], *P*=0.957).

**Figure 3:**
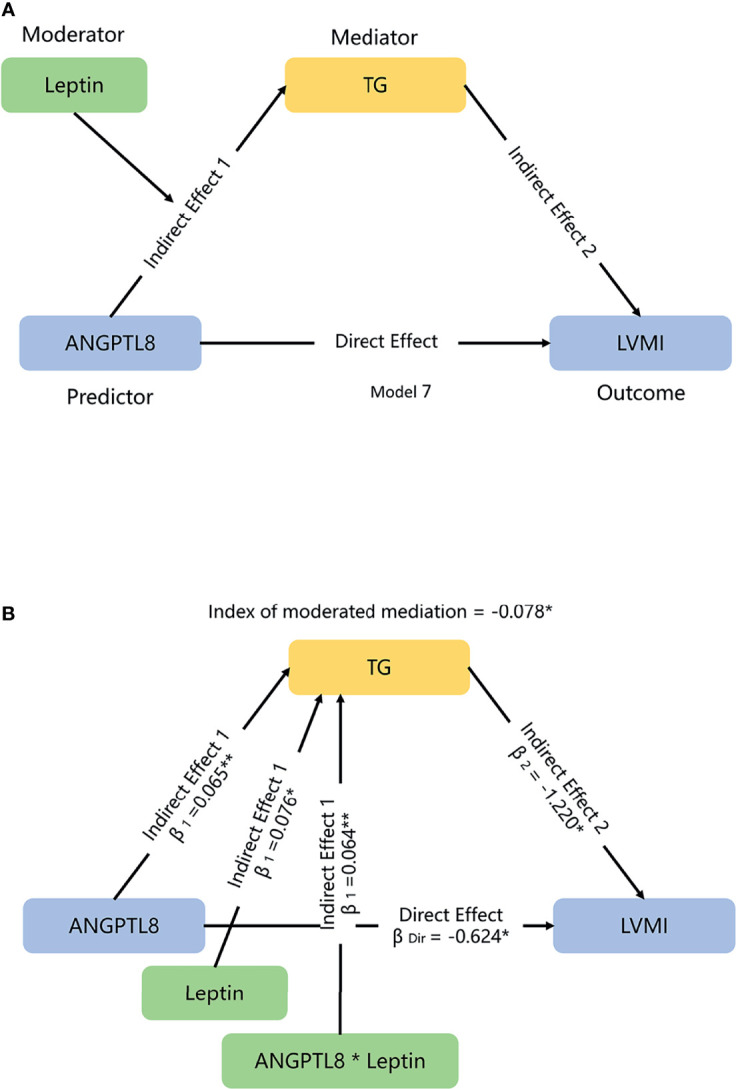
The moderated-mediation model used to evaluate the effects of leptin and TG on ANGPTL8-LVMI association. **(A)** We hypothesized that leptin might modulate the mediation effects of triglyceride (TG) on the association of ANGPTL8 and left ventricular mass index (LVMI) in a moderated mediation template model 7. **(B)** Moderated effect of leptin for TG is significant, with increment of 0.064 mmol/L TG each additional ln-leptin z-score; the index of moderated mediation, which qualify the relationship between moderator leptin and the size of the indirect effect of ANGPTL8 on LVMI through mediator TG, was significant, with a 0.078 g/m^2.7^ lower LVMI each additional ln-leptin z-score at a given level of ANGPTL8. Mediation and moderation effects were adjusted for age, sex, body mass index, and systolic blood pressure. β =standardized regression coefficient; β_1_ = indirect effect 1; β_2_ = indirect effect 2; β_Dir_ = direct effect. Data based on 5000 bootstrap samples. Ln-transformed for TG, ANGPTL8 and leptin, per z-score interaction of each ln-adipokine/hepatokine. *P < 0.05; **P < 0.01.

Second, we explored how much leptin affected the ANGPTL8-LVMI relationship through modulating TG levels. As shown in **Figure 3**, we used leptin level as a moderator and TG as a mediator to explore ANGPTL8-LVMI relationship by using a moderated mediation template Model 7. As expected, we observed a significant interaction between ANGPTL8 and leptin for TG levels, with a 0.064 mmol/L higher ln-TG with each additional ln-leptin z-score (*P* =0.017), and the index of moderated mediation was significant, with a 0.078 g/m^2.7^ lower LVMI each additional ln-leptin z-score at a given level of ANGPTL8 (95% CI: -0.202 to -0.003). **Table 3** showed the conditional indirect effects of ANGPTL8 on LMVI through TG at three levels of leptin (mean ± 1 SD). At high levels of leptin (1SD above mean), we observed a significant indirect effect with a 0.156 g/m^2.7^ lower LVMI for each additional sex-adjusted ln-ANGPTL8 z-score (95% CI: -0.364 to -0.007) through increasing circulating TG, accounted for 20% of the total effect. In contrast, this effect was totally absent at low levels of leptin (1SD below the mean). These results indicate that the significant mediation effect of TG on ANGPTL8-LVMI relationship was largely depending on the leptin levels.

**Table 3:** Conditional indirect effects of ANGPTL8 on LMVI through TG by a moderated-mediation PROCESS model.

Leptin levels	β (SE)	95%CI	Conditional proportion mediated (% of total effect)
Low	0.002 (0.043)	-0.089 to 0.095	/‡
Medium	-0.079 (0.051)	-0.197 to 0.002	11.2
**High**	**-0.156 (0.093)**	**-0.364 to -0.007**	20.0*

Natural ln-transformed for triglyceride (TG), ANGPTL8 and leptin; per 1-SD increment of ln-leptin z-score. The moderator leptin was showed at three different values, mean-SD, mean, and mean ± SD. Conditional indirect effects of ANGPTL8 on LVMI through TG at three levels of leptin (mean ± 1SD) were estimated in a formula: (indirect effect 1 (β_1_) of ANGPTL8 + indirect effect 1 (β_1_) of ANGPTL8*Leptin interaction) multiply with indirect effect 2 (β_2_) in the moderated-mediation Model 7 (**Figure 3**); total effect was estimated in a formula: indirect effect 1 (β_1_) multiply with indirect effect 2 (β_2_) + direct effect (β_Dir_). Data based on 5000 bootstrap samples. Mediated effect was adjusted for age, sex, body mass index, and systolic blood pressure./‡, the proportion mediated is undefined if the mediated effect is not in the same direction as the total effect. Values in bold are significant at P < 0.05.

*P < 0.05.

## Discussion

In the current study of young participants with mean age of 20.2 years recruited from the cohort of BCAMS, we systematically explored the association between cardiac geometry and five prominent adipokines/hepatokines, including ANGPTL8, leptin, adiponectin, FGF21 and RBP4. We observed an inverse association of ANGPTL8 with LVMI in Chinese youths at risk of MS independent of classical cardiovascular risk factors, suggesting a protective effect of ANGPTL8 on cardiac remolding. In addition, while we replicated the negative association between leptin and LVMI, we further found that leptin significantly moderated the ANGPTL8-LVMI association through the mediation effect of TG, suggesting an interaction between adipokine leptin and hepatokine ANGPTL8 on cardiac remolding. Notably, we also found a significant association of other classical adipokines like adiponectin and the hepatokine FGF21 with cardiac parameters, but these associations were not independent of known factors affecting their levels, including sex and BMI.

Although numerous studies have assessed the relation of ANGPTL8 to risk of cardiometabolic diseases (22, 24, 25, 40), previous work has not addressed the relation to cardiac remodeling. Cardiac remodeling, characterized by left ventricle geometry disruption, myocardial hypertrophy etc., is usually accompanied by an imbalance between lipid uptake and β-oxidation of FAs, leading to an accumulation of lipids (41, 42). Given that dysfunction of lipid metabolism is involved in cardiac remodeling, and ANGPTL8 plays an important role in lipid metabolism, we explored whether lipids played a mediation role in the association between ANGPTL8 and cardiac parameters by using mediation analysis. As expected, we found that total cholesterol levels, probably mainly LDL-C, significantly mediated the ANGPTL8-LVMI association, while the mediation effect of TG on this association was conditional on leptin, which was only the case for those participants with high leptin levels. Further investigation is needed to confirm and expand to understanding the pathological mechanisms underlying these observations.

**Figure 4:**
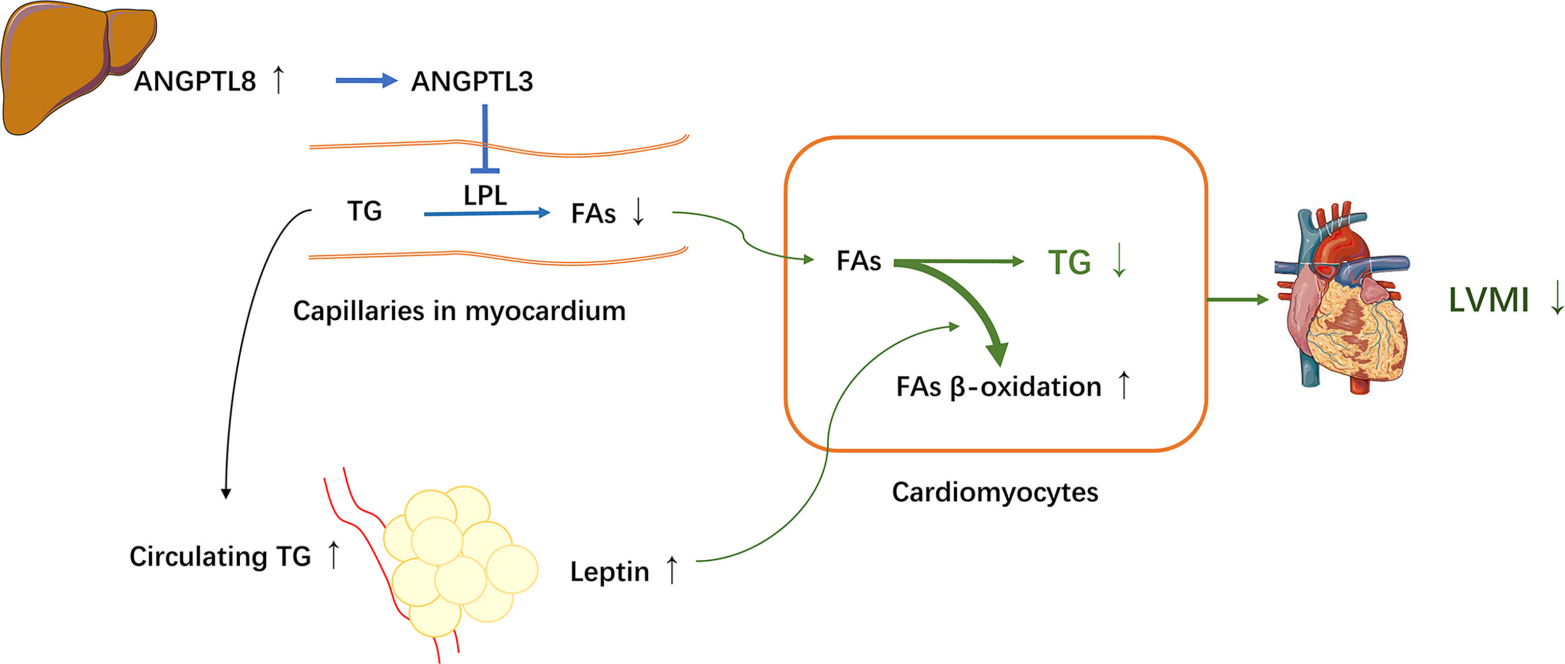
A proposed mechanistic model for interaction between ANGPTL8 and leptin on LVH through triglyceride. The hepatokine ANGPTL8 might facilitate angiopoietin-like protein 3 (ANGPTL3) to inhibit lipoprotein lipase (LPL) activity for conversion of triglyceride (TG) into fatty acids (FAs) in myocardium, resulting in increased circulating TG and decreased FAs uptake into cardiomyocytes, and coordinating the trafficking of TG to white adipose tissue for storage, and ultimately, inducing higher leptin secretion. Meanwhile, high leptin levels might stimulate β-oxidation of FAs and inhibit *de novo* lipogenesis in cardiomyocytes to further prevent myocardial TG accumulation and lipid cardiomyopathy, leading to a favorable LVMI. Thereby, the adipokine leptin and the hepatokine ANGPTL8 cooperate together to decrease cardiac TG involved in mechanism of protective effect on cardiac remodeling.

ANGPTL8 is primarily expressed mostly in the liver and adipose tissue (in mice), and its level is suppressed by fasting and highly induced by feeding, thus it is also known as refeeding-induced fat and liver (RIFL) (25, 43). In addition, ANGPTL8 has also been called lipasin for its LPL inhibition effect (25, 44). LPL is mainly expressed and active in adipose tissue, cardiac and skeletal muscle, acting as a rate-limiting enzyme for hydrolyzing TG presenting in circulating lipoproteins, generating free FAs that are taken up by these peripheral tissues (45). Recent studies have showed that ANGPTL8, together with ANGPTL3 and ANGPTL4, regulates TG metabolism by inhibiting the LPL activity and collaborates to balance the distribution of circulating TG between adipose tissue and oxidative tissues (e.g., cardiac and skeletal muscles) under different nutritional statuses (24). ANGPTL8 has been observed to facilitate ANGPTL3 to inhibit LPL in cardiac and skeletal muscle and coordinate the trafficking of TG to white adipose tissue for storage in response to food intake. During fasting, ANGPTL8 coordinates with ANGPTL4 to inactive LPL in white adipose tissue to direct TG to cardiac and skeletal muscles to supply energy (24, 25, 46, 47). These previous studies indicated that ANGPTL8 plays an important role in TG metabolism, and thereby may contribute to the pathogenesis of cardiac dysfunction. However, in the current study, although the serum TG level was positively associated with ANGPTL8, in line with previous studies, it is surprising that unlike total cholesterol, a significant mediation effect of TG on the ANGPTL8–LVMI relationship was not detected using the simple mediation model. We sought to assess if this may be due to confounding by other factors that may influence the association between ANGPTL8 and LVMI. Leptin has been previously reported to protect against cardiac hypertrophy *via* attenuating cardiac TG accumulation (12, 14). Moreover, in the current study, when leptin was taken into account, the association between ANGPTL8 and LVMI appears more significant in multivariate regression analyses. Therefore, we proposed that leptin may be a moderator influencing the effects of ANGPTL8 on cardiac structure *via* TG. As expected, by using a moderated mediation model, we found that the significant mediation effects of TG on ANGPTL8-LVMI relationship was largely dependent on leptin levels. In such a way the indirect effect of TG accounted for 20% of the total effect of ANGPTL8-LVMI relationship among those participants with high leptin levels, whereas at low leptin levels, this effect was totally lost. Our results suggested that leptin and ANGPTL8 may cooperate together to decrease the deposition of TG in cardiomyocytes, and are hence involved in the mechanism of protective effects on cardiac remodeling. According to our observations and prior experimental studies (12, 14, 24, 25, 46, 47), a proposed mechanistic model of interaction between the hepatokine ANGPTL8 and the adipokine leptin on cardiac structure *via* TG is depicted in **Figure 4**. The increased ANGPTL8 levels might facilitate ANGPTL3 to inhibit LPL activity for conversion of TG into FAs and thus decrease uptake of FAs into cardiomyocytes, resulting in increased circulating TG and the trafficking of TG to white adipose tissue for storage, and ultimately, inducing higher leptin secretion. Meanwhile, high-circulating leptin levels might stimulate β-oxidation of FAs to further prevent myocardial TG accumulation and lipid cardiomyopathy (12, 14), yielding a favorable effect on cardiac structure. However, further studies are needed to directly test and verify these hypotheses.

Another interesting finding is that total cholesterol showed a significant mediation effect on ANGPTL8-LVMI relationship, accounting for 19.0% of total effect, and the effect mediated by cholesterol may largely attributable to LDL-C, which alone accounted for 17.1% of total effect. However, there is no obvious evidence until now to prove the beneficial effect of ANGPTL8 on cardiac remodeling *via* elevating circulating cholesterols. Prior clinical study has found that antibodies-induced ANGPTL3 inhibition resulted in substantial reductions in LDL-C levels in patients (48), and in mice models, researchers have found that ANGPTL3 inhibition promotes VLDL processing and clearance, and lowers LDL-C through inhibition of endothelial lipase (49). Thus, it seems that increased ANGPTL8 levels may activate ANGPTL3, subsequently promote the inhibiting effect of ANGPTL3 on endothelial lipase, leading to increased circulating LDL-C levels. However, it is unclear how elevating circulating LDL-C levels could be a link between ANGPTL8/ANGPTL3 and cardiac remodeling, further mechanistic investigations along these lines will be warranted. Notably, a recent clinical study showed that elevated ANGPLT8/betatrophin levels were associated with cholesterol efflux capacity (50). Since cholesterol efflux capacity has been demonstrated to be reversely associated with the incidence of cardiovascular events in several population-based studies (51, 52), the proposed function of ANGPTL8 in cholesterol transport may provide another possible explanation for the protective role as noted above. However, since there was only one study described the relationship of ANGPTL8 and cholesterol efflux capacity to our knowledge (50), more investigations are needed to verify these findings.

Taken together, in the present study, we found that high ANGPTL8 was associated with smaller LVM, which was significantly mediated by lipids. These results provided insights into the metabolic mechanism relevant to LVH. Nonetheless, it should be noted that either TG or TC can only explain a moderate amount of the total effect of ANGPTL8 on LVMI, suggesting the possibility that other mediators may substantially account for the protective role. In our previous analysis in the same population (28), we found that vitamin D modifies the associations of circulating ANGPTL8 with certain classical cardiometabolic risk factors, but vitamin D levels had no impact on the association between ANGPTL8 and LVMI in current analysis (*P* for interaction = 0.69, data not shown). However, in line with us, a recent prospective study included 533 elderly Caucasian patients with an eight-year follow-up, reported firstly that high ANGPTL8 level protects coronary patients from cardiovascular events and high ANGPTL8 is a valuable biomarker for lowered cardiovascular event risk (53). These findings appear to lend support to our results; however, the underlying mechanism was also not clearly interpretated, and further studies are needed to provide more evidence.

In addition to our novel finding suggesting that leptin interacts with ANGPTL8 to decrease myocardial lipid deposition, which may lead to lower LVMI, evidence from experimental studies has shown that leptin has a variety of protective effects on the heart (14, 54). In human studies, leptin was one of the most studied adipokines in relation to cardiac structure and function in various populations, but the results remain controversial (14), as some studies reveal a protective association (10, 11), while others do not (55–57). Determining the relationship between leptin and cardiac remodeling is often confounded by the known positive relationship leptin and adiposity. In current study, leptin was positively associated with LVMI before BMI was adjusted, while it became negatively related to LVMI when BMI (the indicator of total fat), and even waist circumference (an estimate of visceral and subcutaneous abdominal fat mass) or hip circumference (an estimate of gluteofemoral fat mass) (58) (data not shown) were adjusted. Since leptin was a circulating biomarker of fat content and obesity associated with hyperleptinemia and leptin resistance, while higher adiposity was strongly associated with greater LVMI, it is not difficult to understand that when adiposity is not taken into account, the positive correlation between leptin and LVMI may reflect the relationship between obesity and LVMI; conversely, when the BMI was adjusted, the reverse relationship between leptin and LVMI indicated that at any given degree of adiposity, the higher the leptin and the lower the LVMI, supporting an independent beneficial role of leptin on cardiac remodeling. Thereby, apart from the differences in race, age, cardiac measures, presence of comorbidities (10, 11, 55–57) and no prior data considering the interaction with ANGPTL family, conflicting results in population studies might be at least partly explained by the confounding role of adiposity (59).

Like leptin, adiponectin was also the most frequently examined adipokines relevant to cardiovascular disorders (14). Adiponectin is a collagen-like protein synthesized in white adipose tissue and human cardiocytes, its receptors are expressed in cultured cardiac myocytes (60). It has anti-inflammatory and insulin-sensitizing properties, and is protective against obesity and obesity-related disorders (61). As expected, previously many studies observed an inverse relationships of circulating adiponectin concentrations with LVH, suggesting a protective role on cardiac remodeling (16), However, similar relationship can be observed in our study when BMI was not adjusted (**Supplementary Table 1**), but this association was totally ablated after controlling for BMI, which was in line with the recent report from Framingham Heart Study (11), suggesting that these relationships were highly confounded by adiposity (61).

FGF21 is a member of the FGF superfamily that is produced by the liver, adipose tissue, and skeletal muscle. It stimulates glucose uptake into adipocytes, increases thermogenesis, energy expenditure, fat utilization, and improves glucose and lipid metabolism (7), and the pharmacological benefits of FGF21 in obesity-related metabolic complications, including cardiovascular disorders, has been a hot topic (62). Studies in animal models have found that the FGF21 deficiency is associated with cardiac alterations, including signs of hypertrophy (19), suggesting a protective effect on the heart. In human, the relationship between FGF21 and cardiovascular disease has been well established (7). However, to our knowledge, only two previous studies included elderly patients have assessed the association of FGF21 with cardiac remodeling (20, 21). One study included 95 elderly patients (aged 74.5 ± 11.2 years) with heart failure reported that increased FGF21 was associated with diastolic dysfunction (20), while another reported that high FGF21 level was significantly correlated with left ventricular systolic dysfunction and tended to suffer greater risks of cardiac death in an elderly Chinese population (aged 66.3 ± 10.1 years) (21). Those data indicated that increased FGF21 level was associated with adverse cardiac alterations, suggesting the presence of FGF21 resistance or a compensatory response to the underlying metabolic disturbance or tissue injury may likely be involve in old patients (62). In our study in young population, we also found that higher FGF21 was associated with greater LVM, but this relationship was dependent on BMI, suggesting the link between FGF21 resistance and cardiac remodeling in adolescents and young adults may largely be mediated by adiposity.

As to RBP4, researches exploring whether it has an effect exist on cardiac remolding are very limited. RBP4, originally characterized as a transport protein for retinol to the tissue, is associated with lipid metabolism, and insulin resistance (7, 21). Strong animal experimental data suggested that RBP4 is causally involved in the etiology of cardiometabolic diseases (7, 18). In a recent report from Framingham Heart Study, researchers found that LVMI was positively related to RBP4 adjusting for known correlates (including weight) (11). However, in our study, the association between RBP4 and cardiac geometry was not statistically significant after adjustment of age and sex. These discrepancies might be explained by the differences of different populations or ethnic groups, and varied control for confounders.

Strengths of this study include a relatively large cohort, the standardized protocol with detailed and well-characterized cardio-metabolic phenotype, and the application of statistical methods designed to assess mediation, leading to the finding that high ANGPTL8 level was associated with favorable cardiac structural changes through lipids, and its interaction with leptin. Despite the strength, there are some key limitations to our study. Firstly, the sample was drawn from youths at risk of cardiovascular diseases; future replication study is needed to evaluate the generalizability of our findings to older adults and other ethnical populations. Secondly, we found a negative relationship between ANGPTL8 and abnormal cardiac geometry, but causality is difficult to infer due to the cross-sectional nature of the study. Our ongoing follow-up observation and/or experimental studies are required to elucidate the causal relationship between ANGPTL8 and cardiac abnormality. Finally, although we have included 5 functionally prominent adipokines/hepatokines in our study, we are aware that many others, such as fetuin-A (63, 64), may be implicated in obesity-related LVH. Thus, further researches with more adipokines/hepatokines are needed to figure out the role of tissue-tissue cross-talk in the pathogenesis of LVH.

In conclusion, we found higher ANGPTL8 was associated with smaller LVM in Chinese youths at risk of MS independent of BMI and other classical cardiovascular risk factors, suggesting that hepatokine ANGPTL8 may has a beneficial effect on cardiac remolding in youth with risk for MS; moreover, we observed the adipokine leptin has a moderation effect on ANGPTL8-LVMI relationship through TG. These observations of tissue-tissue cross-talk might offer insights into the pathogenesis of the cardiomyopathy of youths with risk for MS. Further study is needed to assess the prognostic and therapeutic implications of these observations, and clarify the precise underlying mechanism(s) involved.

## Data Availability Statement

The original contributions presented in the study are included in the article/**Supplementary Material**. Further inquiries can be directed to the corresponding authors.

## Ethics Statement

The study protocol was approved by the Ethics Committee at the Beijing Chaoyang Hospital and was in accordance with the declaration of Helsinki on ethical principles for medical research involving human subjects. Written informed consent was obtained from all patients before participation in this study. The study was registered on www.clinicaltrials.gov (NCT03421444). Written informed consent to participate in this study was provided by the participants’ legal guardian/next of kin.

## Author Contributions

DW and DF analyzed the data and wrote the manuscript. YuW, PD, YoW, LZ, BL, and JF contributed to data collection. XX contributed to the data analysis and reviewed the manuscript. JS contributed to the data interpretation and reviewed the manuscript. SG was responsible for the concept, design, and data collection in the BCAMS follow-up study. ML was responsible for the biomarker study of BCAMS, and contributed to acquisition and interpretation of the data, and revised the manuscript. All authors contributed to the article and approved the submitted version.

## Funding

This work was supported by grants from National Key Research program of China (2016YFC1304801), National Natural Science Foundation of China (81970732), Capital’s Funds for Health Improvement and Research (2020-2Z-40117), Beijing Natural Science Foundation (7172169), key program of Beijing Municipal Science & Technology Commission (D111100000611001, D111100000611002), Beijing Science & Technology Star Program (2004A027), Novo Nordisk Union Diabetes Research Talent Fund (2011A002), National Key Program of Clinical Science (WBYZ2011-873), the Non-profit Central Research Institute Fund of Chinese Academy of Medical Sciences (2017PT32020, 2018PT32001), the CAMS Innovation Fund for Medical Sciences (CIFMS, 2021-1-I2M-016)and Key projects of medical school development of Shijingshan district (Beijing).

## Conflict of Interest

The authors declare that the research was conducted in the absence of any commercial or financial relationships that could be construed as a potential conflict of interest.

## Publisher’s Note

All claims expressed in this article are solely those of the authors and do not necessarily represent those of their affiliated organizations, or those of the publisher, the editors and the reviewers. Any product that may be evaluated in this article, or claim that may be made by its manufacturer, is not guaranteed or endorsed by the publisher.
