# Supplementary material for: Angiopoietin-Like Protein 8/Leptin Crosstalk Influences Cardiac Mass in Youths With Cardiometabolic Risk: The BCAMS Study
Source: Front Endocrinol (Lausanne). 2022 Jan 25;12:788549. doi: 10.3389/fendo.2021.788549 (PMC8821093; doi:10.3389/fendo.2021.788549)
Supplement: Supplementary file 1 [file DataSheet_1.docx]

Supplementary Material

# Supplementary Tables

## Supplementary Figures


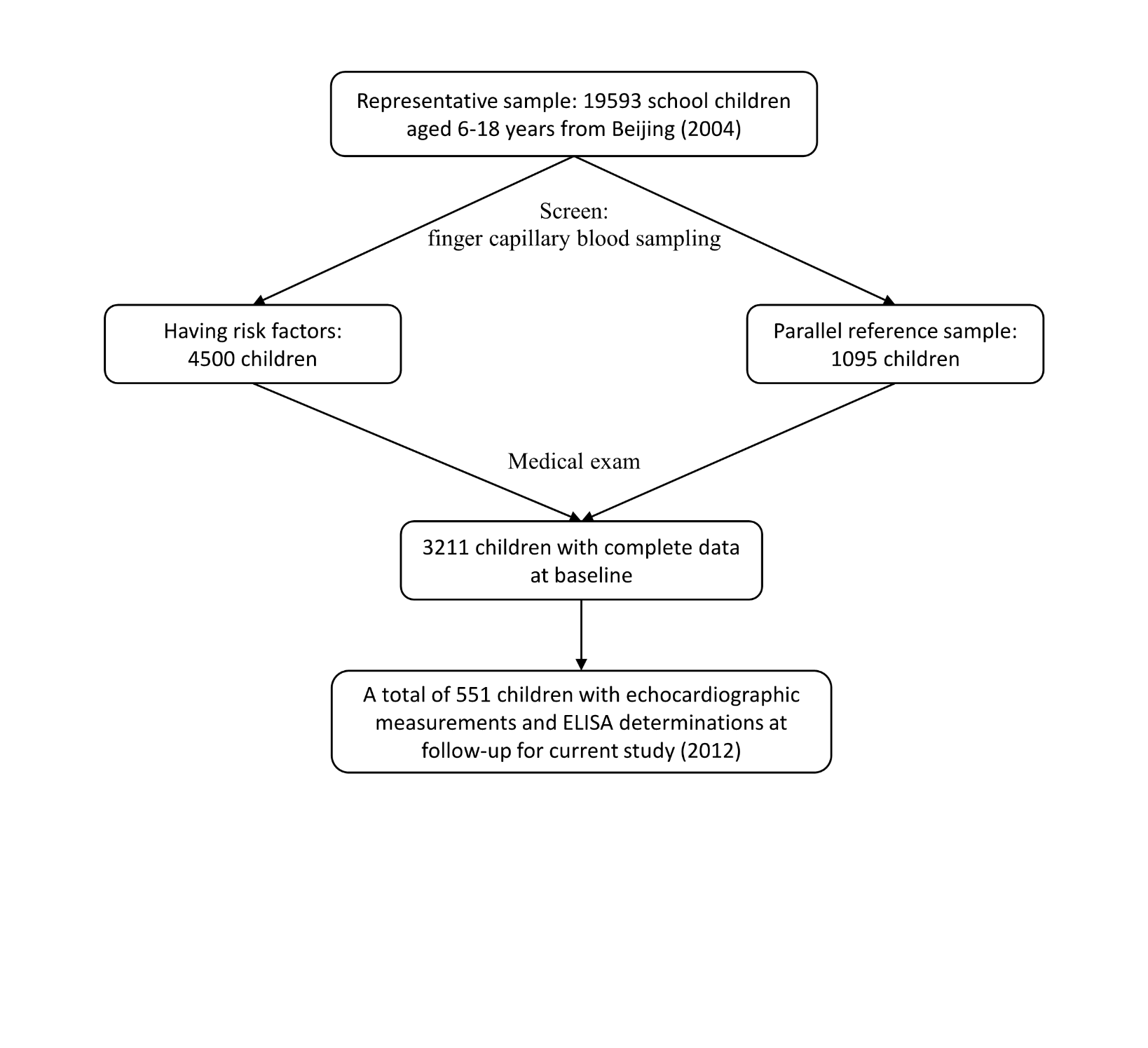


**Supplementary Figure 1.** Flow chart of the cohort.


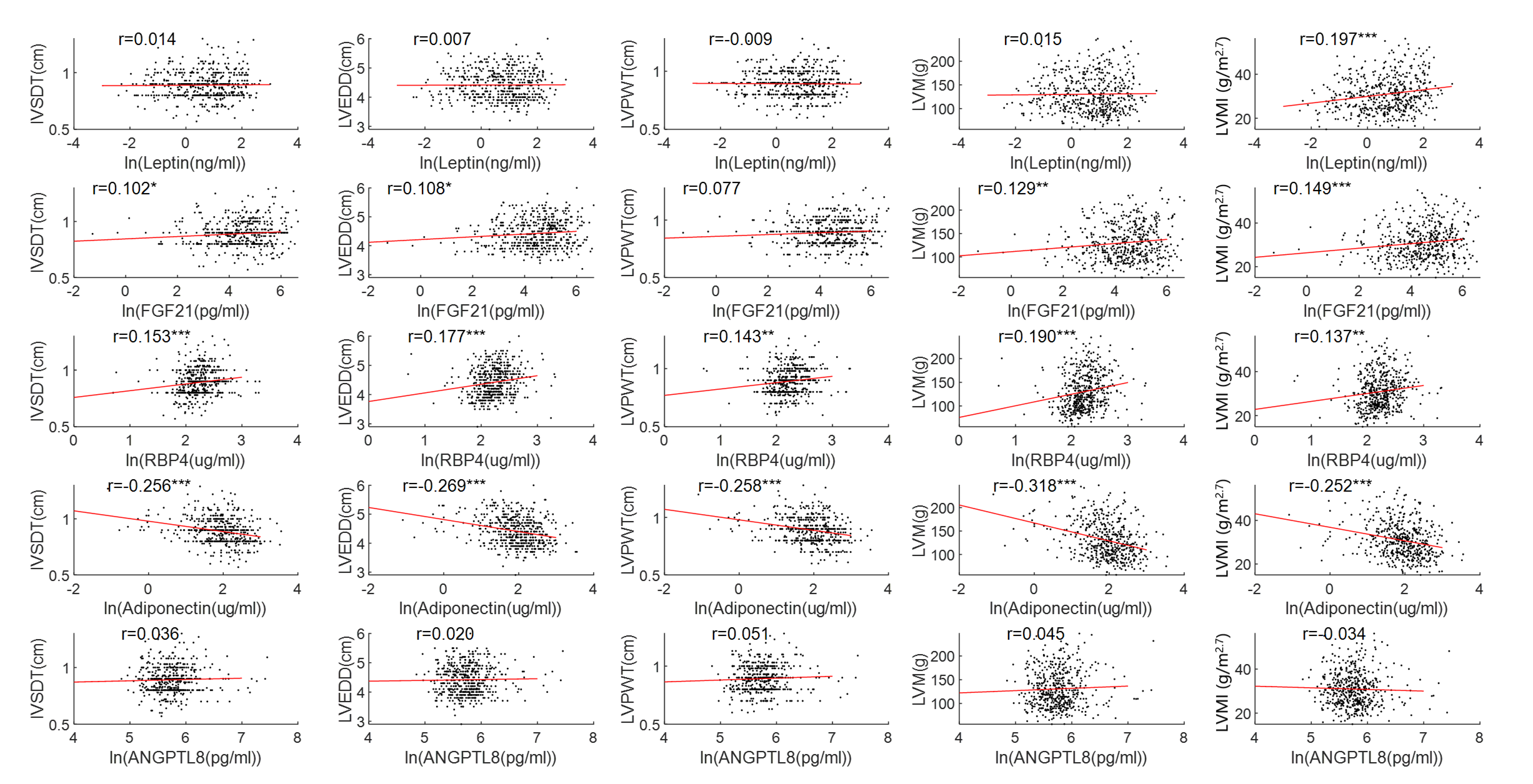


**Supplementary Figure 2.** Scatter diagrams of adipokines/hepatokines related to cardiac parameters without correction of body weight. * *P* <0.05; ** *P* <0.01, *** *P* <0.001. FGF21, fibroblast growth factor 21; ANGPTL8, angiopoietin-like protein 8; RBP4, retinol binding protein 4; APN, adiponectin; IVSDT, inter ventricular septal diastolic thickness; LVEDD, left ventricular end-diastolic diameter; LVPWT, left ventricular posterior wall thickness; LVM, left ventricular mass; LVMI, left ventricular mass index.

## Supplementary Tables

**Supplementary table 1.** Pearson correlation (r) between adipokines/hepatokines and cardio-parameters.

| **Unadjusted** | IVSDT (cm) | LVEDD (cm) | LVPWT (cm) | LVM (g) | LVMI (g/m^2.7^) |
| --- | --- | --- | --- | --- | --- |
| Leptin (ng/ml) † | 0.014 | 0.007 | 0.009 | 0.015 | **0**.**197***** |
| FGF21 (pg/ml)† | **0.102*** | **0.108*** | 0.077 | **0.129**** | **0.149**** |
| RBP4 (ug/ml)† | **0.153***** | **0.177***** | **0.143**** | **0.190***** | **0.137**** |
| APN (μg/ml)† | **-0.256***** | **-0.269***** | **-0.258***** | **-0.318***** | **-0.252***** |
| ANGPTL8 (pg/ml)† | 0.036 | 0.020 | 0.051 | 0.045 | -0.034 |
| **Adjusted for age and sex** | | | | | |
| Leptin (ng/ml) † | **0.238***** | **0.299***** | **0.249***** | **0.355***** | **0.350***** |
| FGF21 (pg/ml)† | **0.104*** | **0.130**** | **0.093*** | **0.158***** | **0.164***** |
| RBP4 (ug/ml)† | 0.048 | 0.051 | 0.059 | 0.062 | 0.078 |
| APN (μg/ml)† | **-0.193***** | **-0.183***** | **-0.179***** | **-0.238***** | **-0.215***** |
| ANGPTL8 (pg/ml)† | -0.045 | -0.073 | -0.041 | -0.067 | -0.084 |
| **Adjusted for age, sex, BMI and SBP** | | | | | |
| Leptin (ng/ml) † | -0.053 | -0.044 | **-0.090*** | -0.077 | **-0.099*** |
| FGF21 (pg/ml)† | -0.025 | -0.013 | -0.055 | -0.024 | -0.015 |
| RBP4 (ug/ml)† | -0.014 | -0.021 | -0.010 | -0.029 | -0.010 |
| APN (μg/ml)† | -0.053 | -0.008 | -0.017 | -0.029 | 0.005 |
| ANGPTL8 (pg/ml)† | -0.060 | **-0.094*** | -0.059 | **-0.097*** | **-0.117**** |

* *P* <0.05; ** *P* <0.01, *** *P* <0.001. BMI, body mass index; SBP, systolic blood pressure; FGF21, fibroblast growth factor 21; ANGPTL8, angiopoietin-like protein 8; RBP4, retinol binding protein 4; APN, adiponectin; IVSDT, inter ventricular septal diastolic thickness; LVEDD, left ventricular end-diastolic diameter; LVPWT, left ventricular posterior wall thickness; LVM, left ventricular mass; LVMI, left ventricular mass index. † Ln-transformed. Values in bold are significant at *P* < 0.05.

**Supplementary Table 2.** Characteristics of the study population according to sex-standardized ANGPTL8 quartiles.

| **Parameters** | **ANGPTL8** | | | | |
| --- | --- | --- | --- | --- | --- |
|  | **Q1(n = 138)** | **Q2(n = 138)** | **Q3(n = 138)** | **Q4(n = 137)** | ***P*** |
| Male, n (%) | 73 (52.9%) | 73 (52.5%) | 73 (52.9%) | 73 (52.9%) | / |
| Age (years) | 20.5 (3.1) | 20.0 (2.8) | 19.8 (2.9) | 20.3 (2.8) | 0.801 |
| BMI (kg/m^2^) | 25.5 (5.5) | 24.8 (5.3) | 25.8 (6.0) | 26.7 (5.9) | **0.047** |
| SBP (mmHg) | 115 (15) | 113 (12) | 115 (14) | 116 (15) | 0.397 |
| DBP (mmHg) | 72 (11) | 73 (11) | 73 (9) | 74 (11) | 0.433 |
| Fasting blood glucose (mmol/L) | 4.86 (0.52) | 4.97 (1.12) | 4.89 (0.44) | 4.95 (0.48) | 0.533 |
| Fasting insulin (mIU/L) † | 8.94 (9.03) | 8.11 (5.72) | 9.16 (7.11) | 10.03 (8.90) | 0.241 |
| HOMA-IR † | 0.37 (0.75) | 0.35 (0.70) | 0.43 (0.75) | 0.46 (0.85) | 0.634 |
| ISI_Matsuda_ † | 1.79 (0.65) | 1.83 (0.58) | 1.77 (0.65) | 1.78 (0.70) | 0.880 |
| Triglycerides (mmol/L) | 0.98 (0.53) | 1.02 (0.44) | 1.17 (0.85) | 1.36 (1.22) | **0.001** |
| Total cholesterol (mmol/L) | 4.27 (0.82) | 4.18 (0.82) | 4.31 (0.78) | 4.61 (1.05) | **<0.001** |
| LDL-C (mmol/L) | 2.49 (0.71) | 2.40 (0.73) | 2.49 (0.65) | 2.73 (0.89) | **0.002** |
| HDL-C (mmol/L) | 1.44 (0.29) | 1.42 (0.31) | 1.43 (0.35) | 1.45 (0.34) | 0.874 |
| Vitamin D (ng/ml) † | 2.69 (0.36) | 2.71 (0.41) | 2.64 (0.35) | 2.55 (0.46) | **0.006** |
| Adjusted for age, sex, BMI and SBP (Adjusted means±SEM) | | | | | |
| IVSDT (cm) | 0.90 (0.01) | 0.89 (0.01) | 0.89 (0.01) | 0.89 (0.01) | 0.870 |
| LVEDD (cm) | 4.49 (0.03) | 4.45 (0.04) | 4.38 (0.03) | 4.35 (0.03) | **0.019** |
| LVPWT (cm) | 0.89 (0.01) | 0.90 (0.01) | 0.89 (0.01) | 0.88 (0.01) | 0.509 |
| LVM (g) | 134.31 (2.38) | 132.93 (2.51) | 129.01 (2.38) | 126.38 (2.42) | **0.008** |
| LVMI (g/m^2.7^) | 31.83 (0.56) | 31.50 (0.59) | 30.88 (0.56) | 29.46 (0.57) | **0.016** |

Quartile values of ANGPTL8 are expressed as Q1, Q2, Q3 and Q4 (pg/ml). For female: Q1, 127.5 to 241.7; Q2, 241.8 to 287.0; Q3, 287.0 to 353.5; Q4 > 353.5. For male: Q1, 142.2 to 276.8; Q2, 276.8 to 329.1; Q3, 329.1 to 389.2; Q4 > 389.2. Q1 as the referent quartile. † Ln-transformed. Unadjusted data were expressed as n (%), mean ± standard deviation. BMI, body mass index; SBP, systolic blood pressure; WC, waist circumference; FAT%, body fat percentage; SBP, systolic blood pressure; DBP, diastolic blood pressure; HOMA-IR, homeostasis model assessment for insulin resistance; ISI_Matsuda_,  insulin sensitivity (Matsuda) index; LDL-C, low-density lipoprotein cholesterol; HDL-C, high-density lipoprotein cholesterol; Hs-CRP, high-sensitivity c-reactive protein; IVSDT, inter ventricular septal diastolic thickness; LVEDD, left ventricular end-diastolic diameter; LVPWT, left ventricular posterior wall thickness; LVM, left ventricular mass; LVMI, left ventricular mass index. Values in bold are significant at *P* < 0.05.
